# Can Cartoons Which Depict Autistic Characters Improve Attitudes Towards Autistic Peers?

**DOI:** 10.1007/s10803-019-04318-0

**Published:** 2019-12-11

**Authors:** Carla Simone Engel, Elizabeth Sheppard

**Affiliations:** School of Psychology, University of Nottingham, University Park, Nottingham, NG7 2RD UK

**Keywords:** Autism, Cartoons, Conative component of attitudes, Knowledge, Peers, Vicarious contact

## Abstract

This study aimed to assess the efficacy of two cartoons which depict autistic characters in improving attitudes towards autistic peers in two separate studies. Forty-six children participated in study 1 (4–7 years), and 47 children participated in study 2 (8–11 years). Both the conative (behavioural) component of attitudes and knowledge about autism were measured before and after the cartoon interventions. Knowledge of autism increased after watching the cartoons in both studies but attitudes to autism only improved in study 1. Knowledge was shown to correlate with change in some but not all attitude measures. The findings suggest that cartoons can improve attitudes to autism, but this may depend on how information is presented.

## Introduction

One feature of autism is difficulty in social communication and interaction (APA 2013). Recent research has highlighted that the social difficulties experienced by autistic individuals may not only arise from cognitive and neurological differences but can also be attributed to negative perceptions, judgements and social decisions made by their peers (Sasson et al. 2017; Sheppard et al. 2016). In Sasson et al. (2017) neurotypical adults watched brief videos of autistic and neurotypical participants introducing themselves. They subsequently rated the autistic participants less favourably on a range of personal qualities (such as how attractive or how likeable the person was) as well as reporting reduced intention to pursue social interactions with them, despite being unaware that some of the participants were autistic. Similar negative first impressions have also been observed in relation to autistic children (Grossman 2015). Typically developing adults were presented with short (1–3 s) audio and visual clips as well as stills of high functioning autistic children and typically developing children re-telling a story. Adults who had no previous knowledge of the differences between autistic and non-autistic children, judged the high-functioning autistic children to be socially awkward at a significantly higher rate than their non-autistic peers.

Accordingly, autistic individuals are less involved in social activities in and outside the classroom, often experience bullying, report poor quality friendships, and have fewer reciprocal friendships (e.g. Tonnsen and Hahn 2015). Peer rejection has also been associated with poor school performance, poor retention in postsecondary settings, aggression problems, and depressive behaviour (e.g. Matthews et al. 2015). Conversely, it has been shown that providing autistic individuals with the opportunity to interact with typically developing peers can help improve social outcomes (Ledford and Wehby 2015; Watkins et al. 2017). Interaction with typically developing peers is associated with an improved ability to negotiate positive and negative social situations, better academic achievements, and improved self-esteem and self-concept (Kasari et al. 2011; Boutot and Bryant 2005). However, peer rejection impedes autistic children from benefiting from these interactions. Given the immense impact that peers have on social outcomes for autistic individuals, there is a vital need to examine factors which encourage peer acceptance and promote positive engagement with autistic peers.

The consistent finding that autistic individuals experience increased rates of peer rejection is in line with previous research which indicates that children hold negative attitudes toward intergroup relationships with peers with disabilities as well as those of differing ethnicity and gender (e.g. Abrams et al. 2003; Dunham et al. 2008). Harnum et al. (2007) found that after reading stereotyped scenarios that presented either an autistic child, a child with ADHD (Attention Deficit and Hyperactivity Disorder) and a typically developing child, peers (aged 7–12) showed a significantly higher rate of avoidance/dislike toward autistic individuals. Children were also more inclined to see the autistic child as unlike themselves. This finding was attributed to the way in which children socially categorise their autistic peers, showing stronger tendencies toward assigning more negative traits to outgroup members than are deserved (Gaertner et al. 2016).

Theoretical models of social categorisation of outgroup members, such as intergroup contact theory (Allport 1954), propose increased direct contact with the outgroup as a means to reduce prejudice and stigmatisation (Allport 1954; Wright et al. 1997). While this type of intervention has been successful (Cameron et al. 2007; Rutland and Killen 2015), it is not always feasible (e.g. segregated communities) or appropriate (e.g. in multicultural communities where physical inclusion does not automatically result in friendship) (Vezzali et al. 2014). Studies indicate that children’s attitudes to other people are shaped by direct experiences (interactions), indirect experiences (books, media), and a child’s immediate social group (family members and peers) (Triandis 1971; Turner and Cameron 2016). Therefore, increasing children’s knowledge and experience with the outgroup through indirect means could also help to improve attitudes towards the outgroup. The indirect contact hypothesis (Wright et al. 1997) emanates from intergroup contact theory (Allport 1954), and operates on this basis. It works on two principle mechanisms: *Extended Contact*—the knowledge that an ingroup member has a friendship with an outgroup member; and *Vicarious Contact*—the observation of an ingroup/outgroup friendship (Vezzali et al. 2014). According to Allport’s (1954) initial intergroup contact theory (also Contact Hypothesis), learning about others through intergroup interaction increases the opportunity for groups to recognise the similarities they share (Pettigrew and Tropp 2008).

Drawing on social cognitive theory (Bandura 1986), evidence suggests that vicarious contact, via educational television, can be used as a basis for observational learning and delivering explanatory information. Research has shown that watching educational television programmes in the home can act as an effective intervention across a variety of contexts in which negative outgroup attitudes arise. In a review examining the impact of television as a vicarious contact experience, Browne Graves (1999) found that watching cartoons featuring ethnically diverse characters (e.g. *Superfriends*, *Barney, Magic School Bus and Sesame Street*) led to positive changes in attitudes toward African-American children. Similarly, Bogatz and Ball (1971) reported that White-American pre-schoolers demonstrated more positive attitudes toward African-American and Latino-American peers after watching 2 years of *Sesame Street*, and *Same and Different*, two programmes that celebrate racial diversity and positive intergroup relationships (Vezzali et al. 2014). Other research has shown that elementary school children similarly expressed heightened positive attitudes after watching cartoons which included intergroup friendships (Katsuyama 1997; Vittrup and Holden 2011).

While indirect contact has been shown to improve attitudes to outgroup members in general, it has been shown to ameliorate the conative (behavioural) component of attitudes in particular. Research suggests that attitude comprises three components: affect, behaviour and cognition (Eagly and Chaiken 1998; Schiffman and Kanuk 2004; Spooncer 1992). Accordingly, indirect contact has been shown to specifically affect the component of attitude which relates to behavioural intentions (Gottlieb and Gottlieb 1977). Vezzali et al. (2012) found that vicarious contact through reading intercultural books encouraged a desire to meet and spend time with outgroup peers. They note that this desire in itself lays the foundation for cross-group friendship to take place. This finding is supported by other studies that revealed positive correlations between vicarious contact and enhanced intergroup behavioural intentions (Mazziotta et al. 2011).

Knowledge, reduction of anxiety, and increased empathy and perspective taking, are the three main mediators through which indirect contact operates (Pettigrew and Tropp 2008; Wright et al. 1997). This raises the question as to whether providing explanatory information about members of an outgroup without the context of intergroup contact/friendship is sufficient to improve attitudes towards that group. Studies addressing this question in relation to autism have yielded mixed findings. Some have found that providing explanatory information about autism, through books, cartoons or targeted educational programmes can elicit an improvement in attitudes and behavioural intentions, aid moral development, and reduce stereotyping in typically-developing children (e.g. Cameron et al. 2007; Morton and Campbell 2008; Price et al. 2016). However, in Swaim and Morgan’s (2001) study, which investigated elementary school students’ attitudes towards an autistic peer in a video scenario, providing information explaining autistic behaviours as well as disclosure of diagnosis had no effect on children’s attitudes towards an autistic boy.

The present study utilised the indirect (extended) contact hypothesis (Wright et al. 1997) as a theoretical framework for testing whether educational television programmes featuring autistic characters can act as a means of increasing peer acceptance and promoting positive engagement with autistic individuals. The study investigated whether two different cartoons could positively elevate the conative component of attitudes towards autistic peers in children aged 4–11 years, and whether any change in attitudes was related to knowledge gained from the cartoons. The two cartoons facilitate learning about the outgroup through different means. Therefore, they were examined in two separate studies. The first cartoon, *Sesame Street,* aimed at 4–7-year olds, operates on the vicarious contact model. It emphasises similarities between viewers and Julia, the autistic character. It depicts intergroup friendships, as well as providing explanatory information about her behaviour. The second cartoon, *Arthur*, highlights the differences and difficulties faced by an individual with Asperger’s syndrome. It relays an informative narrative of Asperger’s syndrome but displays no explicit cross-group friendships. As the two cartoons also target different age groups, it consequently allowed the investigation of both a younger audience (4–7-year-olds) with *Sesame Street*, and an older audience with *Arthur* (8–11-year-olds). Both knowledge and attitudes to autism were measured. Finally, as some earlier studies have revealed mixed results regarding the existence of gender differences in children’s attitudes towards peers with disabilities (e.g. Campbell et al. 2004; de Boer et al. 2012; Swaim and Morgan 2001), the present research aimed to assess whether males and females performed differently. It was predicted that children would have more knowledge about autism after watching the cartoons. Additionally, it was hypothesised that attitude ratings towards an autistic peer would increase after watching the cartoons, and that attitudes would correlate positively with knowledge.

## Study 1

Study 1 examined the impact of *Sesame Street’s* new character Julia, an autistic 4-year-old. The introduction of this character aimed to educate children about the differences and similarities they share with an autistic peer (Westin, EVP of global social impact and philanthropy at Sesame Workshop, as cited in *Guardian* 2017). The character was developed following input from educators, psychologists and activists, as well as personal experiences from staff who have autistic children and siblings (Suskind 2017). The impact of this particular character has not yet been assessed due to her recent debut (April 2017). Therefore, study 1 aimed to explore how much children learned about autism from their first encounter with Julia. Additionally, it aimed to examine whether attitudes and behavioural intentions toward autistic peers improved after watching a short clip of this television show. Participants watched a 3-min segment of the episode. The chosen segment isolates the part of the show explaining characteristic behaviours of autism while also depicting some positive intergroup shared activities. In the segment, Big Bird experiences some difficulty talking to and befriending Julia. Elmo and friends help to highlight her similarities and strengths, as well as explain Julia’s differences regarding her autism.

## Methods

### Design

A 2 × 2 mixed design was employed in which each participant acted as its own control for the within-group variables. A clip of a cartoon featuring an autistic character was used as the intervention. Gender was the between-groups variable. Time of rating attitudes was the within-group variable. Previous knowledge of autism and knowledge gained after watching the cartoon were also measured. The dependent variables were knowledge and attitude ratings.

### Participants

Forty-nine child participants were recruited from Summer Scientist Week (public engagement event) at the University of Nottingham. There were 24 females and 25 males, aged 4–7 years (*M* = 6.34, *SD* = 1.10). Participants’ parents were asked whether their child had any developmental disabilities. One participant was diagnosed with autism and limb girdle muscular dystrophy; one participant was diagnosed with dyslexia, and one participant was diagnosed with mild cerebral palsy which affected their balance. As participants needed to be able to wear headphones, one participant was excluded on the basis that she had a cochlear implant. Two more participants were excluded because they showed visible signs of distraction during the task, leaving 46 participants (23 females; 23 males) for analysis. Most, but not all participants were native English speakers. Those who were not, showed a good command of the English language. Participants were not individually compensated but were given goodie bags at the end of the event. Written informed parental consent was given for all participants. The entire protocol for the study was approved by the University of Nottingham School of Psychology Research Ethics Committee.

### Stimuli and Apparatus

PsychoPy software (Peirce 2007) was used to run the experiment and record participants’ responses using either a mouse or trackpad. Edifier adjustable headphones were used to play all auditory stimuli. A Blusmart lapel microphone and Microsoft voice recording software (Microsoft, Windows 10, 2017) were used to record verbal responses to autism knowledge questions.

The stimuli included four headshots (10 × 16 cm) of two boys and two girls aged 6. Pictures of boys were used for male participants, and pictures of girls were used for female participants. The photos were sourced from various sites using the search engine, Google. Pictures included children of different ethnicities to minimise any other variables that may create ingroup bias. Each child shown in the pictures was given a name. Descriptions of the children were based upon the vignettes of stereotypical autistic children used in Harnum et al. (2007). However, these were adapted to match the characteristics of Julia as described in the cartoon; for example, as follows:

“This is Sarah. Sometimes she does not answer when you talk to her. Usually, she needs you to repeat what you said. When Sarah speaks, she sometimes says the same words over and over again. For example, if Sarah wants to play, she might say “Play. Play. Play.” Sarah doesn’t like the way some things feel when she touches them, and this can upset her.”

As participants had to provide ratings for two different children in the experiment, the order in which these children were presented was counterbalanced, e.g. Sarah then Lucy, or Lucy then Sarah. Their corresponding photographs were counterbalanced in the same way, ensuring that the autistic peer was represented with different ethnicities.

The video was taken from episode 15, ‘Meet Julia’, Season 47, *Sesame Street*, sourced from YouTube. The clip was edited into a 3-min long segment (0:00–3:03) using the iMovie Apple Macintosh application.

Four questions were used to measure the conative component of attitudes, along with an additional question about perceived similarity. The questions were based on the CATCH (Chedoke-McMaster Attitudes Towards Children with Handicaps; Rosenbaum et al. 1986) scale as well as questions used in Sasson et al. (2017) and Harnum et al. (2007) and were formatted as below. The order in which the questions were presented was randomised.

1. How much would you like to play with Sarah?
2. How much would you like Sarah to be your friend?
3. If Sarah were sitting on her own in the classroom, how much do you think you would like to sit with her?
4. How much would you like Sarah to be in your class at school?
5. How similar is Sarah to you?

### Procedure

Participants were told they were going to “meet” a girl/boy around their own age and answer some questions about them. They were also told they were going to watch a short cartoon clip and answer a question about it. They were first presented with instructions and asked if they knew how to use a mouse or trackpad. Those who struggled to use the mouse or did not know how to use it were aided by the experimenter. Next, participants were presented with three test questions to ensure they knew how to use a Likert scale, e.g. ‘How much do you like Broccoli?’ They were given guidelines to indicate what each number on the scale represented, 1 = *Not at all*, 5 = *Very much*. Then participants were presented with a fictional autistic child. A picture of boy or girl around the same age and gender as the participant was displayed on the screen. A description of the child was displayed above the picture and also played via audio recording to ensure those who could not read understood the description. After listening to the description about the boy/girl, participants were told they were going to answer some questions about the child. A set of five questions, which measured the conative component of participants’ attitudes towards autistic peers were presented consecutively on the screen. The experimenter read each question aloud to participants. Participants then responded to each question using the same 5-point Likert scale used in the practice questions. This was a baseline measurement of desire to pursue relationships with autistic peers. Participants were then assessed on their autism knowledge. They were asked ‘What do you know about autism?’ Participants’ answers were recorded, and this used as a baseline measure of their autism knowledge. Participants were then instructed to watch the cartoon clip, making sure that they pay attention and listen carefully. These instructions were presented on the screen and were also read aloud for participants. A short clip of *Sesame Street* was played after which participants were asked, ‘What do you know about autism, now?’. Prompts were used to ensure that participants shared as much knowledge as they had gained from the cartoon clip. Prompts included the questions ‘Do you remember what they said about autism in the video/cartoon?’, ‘Do you remember what they said about Julia?’. Subsequently, participants were told they were going to “meet” another boy or girl. The description of the second child was identical to the first description except it explicitly stated that the boy/girl had autism like Julia. Next, the same set of questions directed toward the second boy/girl were presented on screen. Questions were read aloud, and participants answered on the rating scale accordingly. The experiment took 10–15 min overall.

### Coding

Recorded responses to the autism knowledge questions were coded into two categories: number of statements true of autism before the video, number of statements true of autism after the video. A total of four statements true of autism were supplied in the video, and one non-verbal indication, i.e. hands flapping—hence if a child had no knowledge of autism prior to the video, five would be the maximum score he/she could obtain. Any statements that a child made that were true of autism were given credit regardless of whether they were supplied in the video. Responses were coded by two researchers. Any responses that were coded differently were discussed, and a consensus was reached by the two researchers. Approximately 97% of responses coded were agreed upon; 3% were deliberated. From this, the number of statements true of autism was tallied up for each child before and after watching the video (regardless of whether the statements were prompted or unprompted). Due to the participants being children, researchers took into account limitations in verbal ability when coding true and false statements.

## Results

### Knowledge

Three participants (7%) knew something about autism before watching the video clip. Twenty-five participants (54%, 13 females, 12 male) did not provide any true statements about autism after watching the video clip. Table 1 shows the mean number of true statements participants made about autism before and after watching the video. A Wilcoxon Signed Ranks test showed a significant overall increase in knowledge gained after watching the cartoon *Z *= − 3.731, *p* ≤ 001. Notably, all of the statements made about autism after watching the video directly reflected the content of the video. There was no significant difference in knowledge between males and females, before and after the video.

**Table 1:** Mean number of true statements about autism before and after watching Sesame Street

Gender	Knowledge (before) M(SD)	Knowledge (after) M (SD)
Males	.21 (.66)	.83 (.87)
Females	.00 (0)	.96 (.98)
Total M (SD)	.10 (.47)	.90 (.82)

### Attitude Ratings

**Fig. 1:**
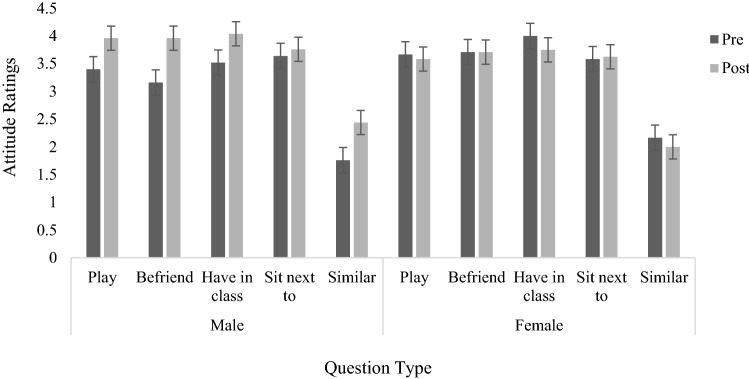
Mean attitude ratings of boys and girls before and after watching the cartoon (error bars indicate standard error)

Figure 1 shows the mean attitude ratings before and after participants watched *Sesame Street* for males and females. Analysis showed that Cronbach’s alpha for the 5-question scale was 0.77, which is considered acceptable to good. Consequently, a mean global attitude rating score was calculated for each participant based on the average of the five items. A 2 × 2 (gender × time) ANOVA revealed a main effect of time, *F*(1,44) = 4.235, *MSe *= 1.73, *p* = .046, *η*_*p*_^*2*^= .088, indicating that ratings given after the video were significantly higher than ratings given before. There was no effect of gender but there was an interaction of Time × Gender, *F*(1,44) = 8.06, *Mse *= 1.73, *p* = .007, *η*_*p*_^*2*^= .155. Simple main effects indicated boys’ ratings increased significantly, *p* = .001, after watching the video, while girls’ ratings did not change.

### Knowledge and Attitude Ratings

Kendall’s tau_b correlations were used to examine the relationship between knowledge (post cartoon) and change in rating for each attitude question, as well as age. Change in rating scores were calculated by subtracting the rating given for the child viewed before the cartoon from the rating given for the child viewed after the cartoon. Table 2 shows the results, with the figures in the bottom row (in bold) indicating the correlations between knowledge and the other variables. Knowledge correlated significantly and positively with age and tended to correlate positively with the attitude questions, although only two of the five were significant: “how much would you like to play with…” and “how much would you like to have … in class”.

**Table 2:** Kendall’s tau_b correlations of attitudes ratings, age, and knowledge (post cartoon)

	Age	Play	Befriend	Sit next to	Have in class	Similarity
Age						
Play	.23					
Befriend	− .05	.18				
Sit next to	.15	.14	.25*			
Have in class	.19	.28*	.37**	.04		
Similarity	− .07	.06	.27*	.17	− .03	
Knowledge	.48**	.26*	**.23**	**.04**	.31*	− **.09**

* significant at *p* < .05, ** significant at *p* < .01. (two-tailed)

## Discussion

The present study aimed to evaluate the influence of *Sesame Street* on the conative component of attitude towards a hypothetical autistic peer. Knowledge gained from the cartoon segment, and the influence of knowledge on attitude ratings were assessed. Consistent with the initial hypothesis, participants showed greater knowledge of autism after watching *Sesame Street*. However, the mean score was below 1, suggesting a limited acquisition of knowledge overall. The cartoon intervention appeared to increase attitude ratings towards autistic peers, consistent with the original hypothesis. However, only boys’ attitudes increased after watching the cartoon, suggesting that the intervention affected boys’ attitudes but not girls. Knowledge correlated positively with age, a desire to play, and a desire to have the autistic peer in their class. This supports the hypothesis that increased autism knowledge would positively correlate with improved attitudes.

## Study 2

Study 2 aimed to assess whether watching the cartoon *Arthur* increased positive attitudes and behavioural intentions towards autistic peers. As in study 1, participants were shown a short segment of the episode, which shows a character called ‘the Brain’ describing what it is like to have Asperger’s syndrome. The segment provides explanatory information, giving examples of the way in which people with Asperger’s syndrome might find the world different, and difficult to navigate but does not depict intergroup friendship. Although *Arthur* is aimed at 4–8-year olds, its content is more mature than most children’s educational programmes. Therefore, it was employed for participants aged 8–11 years. The method was similar to study 1, although some changes were made to accommodate the age of participants and the differing features of the cartoon.

## Methods

### Design

Study 2 employed the same design used in study 1.

### Participants

A convenience sample of 48 participants were recruited from Summer Scientist Week at the University of Nottingham. There were 25 females and 23 males, age range 8–11 years (M = 9.73, SD = 1.06). One participant who was unable to use headphones was excluded from analysis (female, age 9). Participants’ parents were asked whether their child had any developmental disabilities. One participant was diagnosed with autism and ADHD, one participant was diagnosed with ADHD, and one participant was diagnosed with mild dyslexia. Most, but not all participants were native English speakers. Those who were not, showed a good command of the English language.

### Apparatus and Stimuli

Apparatus and stimuli were similar to those employed in study 1. However, headshots included pictures of two girls and two boys aged 10. The cartoon clip was taken from Episode 6, ‘When Carl met George’, Season 13. The clip was 2:18 min long (7:02–9:20). The description of the children shown in the photos was written according to the characteristics of Asperger’s syndrome described in the cartoon clip:

“This is Anna. Anna is very, very smart but she finds it hard to make friends. She has trouble being around other people because sometimes they talk too loudly. Anna also has difficulty understanding what people mean, or when they tell jokes. Sometimes normal things can seem strange to her and this makes her feel different from other children.”

As the main character had Asperger’s syndrome, all knowledge questions used the term Asperger’s syndrome, e.g. ‘What do you know about Asperger’s syndrome?’, and, ‘What do you know about Asperger’s syndrome, now?’

### Procedure

The same procedure from study 1 was employed with some small changes. Most participants were able to read the attitude questions on their own, but the experimenter assisted those who indicated that they wanted help.

### Coding

Coding for knowledge responses was conducted in the same way as study 1. In the *Arthur* clip a total number of eight statements about Asperger’s syndrome were supplied. Hence, this would be the maximum score a child would obtain if they had no prior knowledge of Asperger’s syndrome.

## Results

### Knowledge

Six participants (13%) knew something about Asperger’s syndrome before watching the clip. Eight participants (17%, 3 female, 5 male) provided no true statements about Asperger’s syndrome after watching the clip.

Descriptive data in Table 3 suggest that overall, participants made gains in knowledge after watching the cartoon. Wilcoxon Sign Ranked test indicated a significant increase in knowledge after watching the cartoon *Z* = − 5.071, *p* < 001. There was no gender difference in the level of knowledge on Asperger’s syndrome before or after watching the cartoon.

**Table 3:** Mean number of true statements about Asperger’s Syndrome before and after watching Arthur

Gender	Knowledge (before) M (SD)	Knowledge (after) M (SD)
Male	.36 (.92)	2.13 (1.23)
Female	.21 (.83)	2.58 (1.61)
Total M (SD)	.30 (.87)	2.35 (1.44)

### Attitude Ratings

**Fig. 2:**
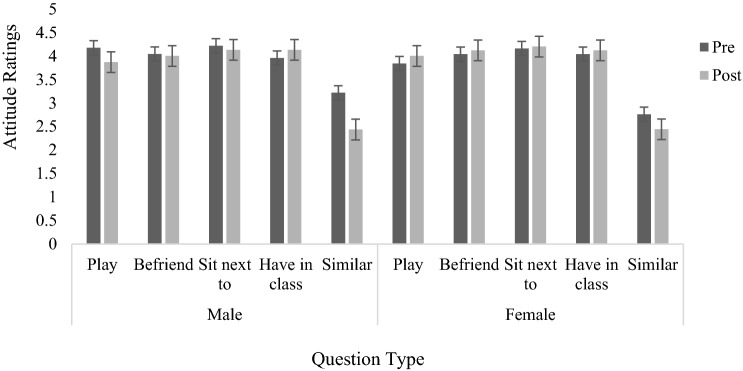
Mean attitude ratings of boys and girls before and after watching the cartoon (error bars indicate standard error)

Figure 2 shows the mean attitude ratings for each question for boys and girls before and after they watched *Arthur*. Analysis showed that Cronbach’s alpha for the 5-question scale was 0.70, which is considered to be acceptable. Consequently, a mean global attitude rating score was calculated for each participant based on the average of the five items. A 2 × 2 (gender × time) analysis of variance gave rise to no effect of time or gender, and no interaction.

### Knowledge and Attitude Ratings

Kendall’s tau_b correlations were conducted to assess the relationship between knowledge gained, attitude ratings, and age. Measurements of knowledge gained and change in attitude ratings were calculated in the same way as study 1. Table 4 shows the results of the analysis, with the figures in the bottom row (in bold) indicating the correlations between knowledge and the other variables. Knowledge correlated significantly and positively with age and tended to correlate positively with the attitude questions, although only two of the five were significant: “how much would you like to play with…” and “how similar is … to you”.

**Table 4:** Kendall’s tau_b correlations of attitudes ratings, age, and knowledge (post cartoon)

	Age	Play	Befriend	Sit next to	Have in class	Similarity
Age						
Play	.12					
Befriend	− .04	.25				
Sit next to	− .15	.31*	.36**			
Have in class	.18	.17	.00	.07		
Similarity	.39**	.26	.23	.17	.08	
Knowledge	.33*	.28*	**.05**	**.02**	**.19**	.31*

Bold indicates correlation with knowledge

* significant at *p* < .05, ** significant at *p* < .01. (two-tailed)

## Discussion

The aim of the present study was to assess the impact of watching *Arthur* on the conative component of attitudes of 8–11-year olds towards a peer with Asperger’s syndrome. In line with the hypothesis, participants showed gains in knowledge after watching the cartoon. Overall attitudes did not significantly increase after watching the cartoon. As predicted, knowledge positively correlated with an increased desire to play with the autistic peer, increased perceived similarity with the autistic peer, and age.

## General Discussion

The present study aimed to examine the efficacy of using a cartoon character to increase knowledge about autism. In both studies, participants knew significantly more about autism after watching the cartoon. The amount of knowledge gained correlated positively with age, suggesting that the older participants are, the more knowledge they gain from such programmes. This finding is supported by developmental research that suggests that children’s ability to grasp important information relayed by educational television develops with age (Fisch and Shalom 2004). This is due, in part, to the acquisition of a myriad of skills such as increased prior knowledge, which allows children to make inferences about information relayed; processing speed; automaticity; and attentional skills (Anderson and Burns 1991; Bandura 1986; Fisch and Shalom 2004). Despite this result, the knowledge participants gained was limited, with only 46% of younger children providing at least one true statement about autism after watching the cartoon. This result was surprising given the fact that testing took place immediately after watching the video: it is likely that memory performance would be even poorer over a longer time period. The poor recall might be partly explained by the way in which knowledge was assessed, which was via a single open-ended question. We chose this format because we wanted to see which aspects (if any) children would spontaneously recall after viewing the cartoons. It is possible that if we had used a different method such as multiple-choice questioning the children might have shown evidence of recalling more of the video content. Given that *Sesame Street* is aimed at pre-schoolers and *Arthur* is aimed at 4–8-year olds, the cartoons should have been easy to understand as most participants were well above the target age group. This begs the question as to whether the content relayed in the cartoons is, in fact, age appropriate.

The second research objective was to examine whether watching the cartoons would positively elevate the conative component of attitudes towards autistic peers, and whether this related to the knowledge participants gained from the cartoons. Evidence is mixed regarding the first point, as attitudes improved in study 1 after watching the cartoon but not in study 2, providing some preliminary evidence in support of the hypothesis. Nevertheless, results from both studies indicated positive correlations between some attitudes and knowledge. In both studies knowledge positively correlated with an increased desire to play with an autistic peer. Additionally, younger participants’ desire to have an autistic peer in their class increased with knowledge. Moreover, in study 2, knowledge was positively associated with perceptions of similarity. These correlations, albeit modest, give some support to the thesis that knowledge can positively influence the desire to pursue social relationships with autistic peers. On the other hand, the fact that only some attitude variables correlated with knowledge and that these correlations were not large may imply that other mechanisms account for attitude change such as reduction of anxiety, and increased empathy and perspective taking (Pettigrew and Tropp 2008; Wright et al. 1997).

Gender also played a role study 1, although not study 2. In study 1, male participants demonstrated an increase in ratings after watching the cartoon, suggesting that males were more receptive than females to the *Sesame Street* segment. This stands in contrast to previous literature which suggests that explanatory information about autism positively elevated female academic behavioural intentions in comparison to their male counterparts (Campbell et al. 2004). This finding is also inconsistent with data from a study assessing the impact of watching *Sesame Street*. which reported a greater impact on girls than on boys (Larkin et al. 2009). The finding in the present study could possibly be attributed to the fact that girls’ ratings started off higher to begin with, perhaps creating a ceiling effect. That girls’ attitudes were quite positive from the outset corresponds with research which suggests that girls tend to express more positive attitudes to those with disabilities (Siperstein et al. 1977). However, given the small sample (n < 25 for each gender), it is necessary to note that these results should be interpreted with caution, and replications should be carried out with larger samples of males and females.

Given the differing effect of the cartoons in study 1 and study 2, it could be that either age, or the manner in which information about autism was delivered, impacted participants’ responses. The forthcoming discussion will examine the potential role of each of these factors. As it has been well documented that younger children express more positive attitudes towards peers with disabilities than adolescents (de Boer et al. 2012; Nowicki and Sandieson 2002), this could suggest that differences in age explain the differences in attitude change. However, this interpretation is at odds with the observation in previous studies that attitudes tend to improve up to adolescence, and only drop off at this stage (de Boer et al. 2012; Ryan 1981; Rosenbaum et al. 1988). Moreover, within the groups in each study, age generally did not correlate with attitude change, suggesting no clear relationship between these factors.

The second possibility is that the different content of the videos used in each study accounts for the difference in results. Perhaps the *Sesame Street* segment represents a truer model of vicarious contact, resulting in more positive results. Along with information about autism, Julia was shown to share activities with the ingroup (painting); her positive attributes were highlighted (‘she’s fun’, ‘she loves to play’); and clear examples of friendship and commonality were depicted throughout the segment. Accordingly, the cartoon presented a depiction of friendship with an autistic peer, and this positive message may have led to elevated positive ratings after watching *Sesame Street*. One can tentatively propose that the observation of cross-group friendship, along with knowledge, positively impacted the conative component of children’s attitudes, supporting the vicarious contact theory. In contrast, the *Arthur* segment articulated the difficulties that a person with Asperger’s syndrome might encounter. Participants were told that people with Asperger’s syndrome feel like they ‘don’t always fit in’, that they don’t always understand people or jokes; that people find them ‘weird’; and that even though people with Asperger’s syndrome learn to fit in they will always feel different. The segment relates a clear message that people with Asperger’s syndrome are different and the attributes assigned to the autistic character were not necessarily positive. The cartoon segment did not emphasise many strengths of the character or many similarities between the character and the viewer. According to indirect contact theory, similarities need to be emphasised to dispel anxiety and promote friendship (Vezzali et al. 2014). In light of this, the fact that attitudes did not improve is not surprising, and in fact perceived similarity showed a numerical decrease after the cartoon.

The result from study 2 echoes that of the Swaim and Morgan (2001) study in which the provision of information about autism had no effect on attitudes towards an autistic peer. The information about autism provided to the children in their study appeared to be similarly negative at times with phrases like ‘Robby has autism, which means that there’s something wrong with his brain’, and ‘He may even hit or bite himself or other people or things’. These results also correspond with earlier research by Siperstein and Bak (1980) which found that when children received lessons about blindness, they expressed increasingly negative attitudes. They proposed that information served to further alienate the blind child as providing it emphasised differences rather than similarities. It seems that the manner in which information about a condition is presented is central to its positive influence. Emphasising differences may lead to further alienation of the autistic children. Information should be delivered in conjunction with clear examples of similarities between ingroup and outgroup as well as an emphasis on the strengths and positive attributes of the outgroup member. Observations of shared activities and intergroup friendship are also important factors contributing to the improvement of attitudes and behavioural intentions in typically developing peers. Further exploration into the delivery of information about autism via diverse forms of vicarious contact interventions in schools, especially at the pre-adolescent stage, is needed to best enhance our understanding of how to improve attitudes and behavioural intentions towards autistic peers.

In relation to the current findings, although it seems more likely that the way knowledge was delivered in each cartoon has primarily led to differing results between studies, future research should consider a design which employs the same intervention across age groups to better clarify reasons for these divergent results. However, this poses a challenge as educational programmes targeted at one age group may not match the comprehension level of another group. Another limitation of the studies is that the videos were just brief excerpts that introduced autistic characters. Typically, in everyday life children will view these videos within the context of a longer show and may have the opportunity for repeat exposure to the same characters over time. Therefore, it is likely that such experiences would result in much greater gains in knowledge, and potentially greater attitude change, than observed in the studies here. However, it is worthy to note that while negative judgements are made quickly, with ‘thin slices’ of clips and stills of autistic individuals (Sasson et al. 2017; Grossman 2015), the results of this study suggest that it may take longer time to overcome these judgments, or to form positive judgements in general. Future research may conduct a study with repeated views, measured at more than two time points, to examine the potential for greater gains in knowledge and attitude change.

A further limitation of the study is that no actual behavioural measure was included. It is possible that children’s responses were affected by socially desirability bias in that they have not felt that it was socially desirable to reveal negative attitudes towards peers. Nevertheless, while this issue could affect the overall level of positivity in their attitudes, it does not seem to explain the change in attitudes that occurred in each study after watching the videos. More generally, it is known that self-reported attitudes do not always predict behaviour strongly, as demonstrated in Lapierre’s (1934) seminal study which suggests that attitudes captured in attitude-questionnaires do not always predict behaviours in specific situations. Therefore, future research should attempt to include a behavioural measure, such as whether or not the participant signs up to volunteers with a local autism organisation (Gardiner and Iarocci 2014), although finding a suitable measure might be challenging with young children.

In summary, children aged between 4 and 11 gained knowledge about autism after watching brief sections of cartoons with autistic characters. Although attitudes towards autism only improved after watching one of the two cartoons, in both studies there were some correlations between knowledge gained and positive change in attitudes. Altogether the findings suggest that knowledge about the condition is useful, but the manner in which it is delivered must be carefully considered.
